# Effects of Surface and Morphological Properties of Zeolite on Impedance Spectroscopy-Based Sensing Performance

**DOI:** 10.3390/s121013284

**Published:** 2012-10-01

**Authors:** Jianwei Zhang, Xiaogan Li, Jeremy White, Prabir K. Dutta

**Affiliations:** 1 School of Electronic Science and Technology, Key Laboratory of Liaoning for Integrated Circuits Technologies, Dalian University of Technology, Dalian 116024, China; E-Mail: jwzhang@dlut.edu.cn; 2 Department of Chemistry, The Ohio State University, 120 W. 18th Avenue, Columbus, OH 43210, USA; E-Mail: jcwhite@chemistry.ohio-state.edu

**Keywords:** chemical warfare agents, zeolite membranes, chemical gas sensors

## Abstract

Measurement by impedance spectroscopy of the changes in intrazeolitic cation motion of pressed pellets of zeolite particles upon adsorption of dimethylmethylphosphonate (DMMP) provides a strategy for sensing DMMP, a commonly used simulant for highly toxic organophosphate nerve agents. In this work, two strategies for improving the impedance spectroscopy based sensing of DMMP on zeolites were investigated. The first one is the use of cerium oxide (CeO_2_) coated on the zeolite surface to neutralize acidic groups that may cause the decomposition of DMMP, and results in better sensor recovery. The second strategy was to explore the use of zeolite Y membrane. Compared to pressed pellets, the membranes have connected supercages of much longer length scales. The zeolite membranes resulted in higher sensitivity to DMMP, but recovery of the device was significantly slower as compared to pressed zeolite pellets.

## 1. Introduction

Developing detection strategies for complex chemical species, such as chemical warfare agents (CWAs) is an active area of research [1–11]. Electrochemical methods are attractive since they have the potential for high sensitivity, miniaturization and low cost. Tomchenko *et al.* tested several metal oxides including SnO_2_, WO_3_, In_2_O_3_ and CuO and found that most of them exhibit good sensitivity to CWAs and their simulants [7–11]. However, Kim *et al.* found that the metal oxide based sensors had poor recovery, possibly due to the strong adsorption of the products from the decomposition of the analyte molecules on the oxide surfaces [10,11]. Also, since these chemoresistive gas sensors use the change in electronic resistance induced by the redox reactions between the adsorbed surface oxygen and “targeted” species, selectivity is often a problem. In addition to the metal oxides, thin-films of metallophthalocyanine (MPc) semiconductors also show promise for CWA detection [6]. Thus, there is a need for development of new sensing materials and transduction mechanisms for detection of CWAs [1,4].

Zeolites are an important group of materials that find practical applications as molecular sieves, catalysts, ion-exchange agents, and gas sensors [12]. In chemical gas sensing applications, earlier work has focused on using zeolites as catalytic filters to improve selectivity [13–15]. Recently, the electrical conductivity of zeolites has been investigated and examined for sensing of volatile compounds [16–22]. Plog *et al.* explored sensing performance of zeolite films on interdigitated electrodes and found that PtNa-zeoliteY as the sensing element indicated good sensitivity to butane [18,19]. Moos *et al.* developed selective NH_3_ sensors by using ZSM-5 [20,21].

In previous work in the authors' laboratory, the use of a pellet type sensor using Na-zeolite Y (abbreviated as NaY) powder as the sensing material for dimethylmethylphosphonate (DMMP), at concentrations of 20–100 ppm was demonstrated [22]. The proposed sensing mechanism involved the interference with transport of sodium cations within the zeolite. The “jumping” rate of the cations was facilitated by the reorientation of the DMMP molecule, leading to the decreased impedance when the sensor is exposed to DMMP. However, several issues with the NaY based sensor needed improvement, including issues with baseline drift and sensitivity [22]. Two strategies are explored in the present paper to address these issues. First, surface modification of NaY particles by ceria was carried out. Second, thin membranes of zeolite Y have been explored for the first time for impedance-based sensing.

## 2. Experimental Section

### 2.1. Material Preparation and Characterization

#### 2.1.1. Synthesis of Ceria Modified Zeolite Y

Sodium zeolite Y (NaY) with Si/Al ratio ∼2.5 was purchased from Zeolyst Int (Conshohocken, PA, USA). Hydrated Ce(NO_3_)_4_ (Aldrich, St. Louis, MO, USA) was dissolved in ethanol and then added to NaY. The mixture was stirred overnight and then heated to 80 °C to evaporate the solvent. Subsequently, the white powders were further calcined at 550 °C for 5 h to decompose the nitrate and form a ceria coating. The NaY-CeO_2_ samples with three compositions, 10, 30 and 50% wt. CeO_2_, were synthesized. For comparison, Ce ion-exchanged into NaY was prepared using aqueous solution. Two grams NaY was added to 200 mL of a 0.01 M aqueous solution of hydrated Ce(NO_3_)_4_ (Aldrich), the resulting suspension was stirred at room temperature overnight, filtered and the procedure repeated a second time.

#### 2.1.2. Fabrication of the Thin Film Zeolite Y

The zeolite Y thin film was fabricated following the procedures described in [23]. In brief, zeolite Y seed layers were deposited on the alumina supports using a dip-coating method. The zeolite Y dip-coating solution was prepared by dispersing 100 mg of the dried, calcined zeolite Y in 20 mL of water, using 2 h of sonication. The 5 mg/mL zeolite suspension was then screened using a 20 micron nylon mesh to remove larger particulates. During dip-coating, the alumina substrate was brought in contact with 16 mL of the screened zeolite suspension in a watch glass at a speed of 0.01 m/s. The seeded alumina was dried overnight in a vacuum oven at room temperature. These alumina supported zeolite seed layers were placed face up in a 125 mL Teflon-lined Parr digestion vessel at a 45° angle in a zeolite Y suspension, filled to 80% of total vessel volume. The zeolite Y suspension was an opaque gel with a molar composition of 17Na_2_O:1Al_2_O_3_:12.80SiO_2_:975H_2_O. This mixture was prepared by mixing 85.24 g water, 2.208 g Al(OH)_3_, and 7.29 g NaOH, which was then added to 13.85 g Ludox SM-30 colloidal silica with vigorous stirring. Upon mixing, a moderately viscous gel is formed which is aged while stirring for 4 h. The autoclave vessel was heated using static conditions at 363 K for 8 h prior to quenching in cold water. Each membrane was then washed well with water and allowed to dry overnight at room temperature. Eventually, the as-prepared thin films were further calcined at 600 °C for 2 h, to remove any templating agent within the pores.

#### 2.1.3. Microstructure Characterization

Powder X-ray diffraction (XRD) patterns were collected on a Rigaku Geigerflex diffractometer using Ni-filtered Cu Kα radiation at 40 kV and 25 mA between 2θ of 20–80° at a scanning speed of 12°/min. Diffuse reflectance infrared spectra (DRIFTS) were collected on zeolite Y powders (3% wt in KBr; using a Perkin-Elmer infrared spectrometer (2 cm^−1^ resolution) equipped with a Pike Technologies diffuse reflectance attachment. All spectra were converted using the Kubelka-Munk transformation with KBr as the reference. Raman spectroscopy was performed using a Renishaw-Smiths Detection Combined Raman-IR Microprobe equipped with an argon ion laser. The zeolite membrane surface morphology was investigated by scanning electron microscopy (SEM) (JEOL JSM-5500, JEOL, Tokyo, Japan and Sirion FEG, FEI Company, Tustin, CA, USA) on gold-coated specimens. In addition, SEM of fracture cross-sections of the supported thin film structure provided information on seed layer and membrane thickness.

### 2.2. Sensor Fabrication and Measurements

**Figure 1.**
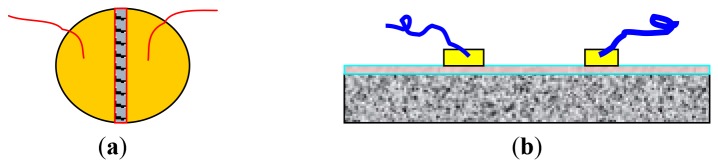
Schematic views of the electrode geometry of (**a**) the pellet-type and (**b**) thin-film type zeolite Y based sensors.

The procedures for fabricating the pellet-type zeolite based sensors are as follows. The NaY and CeO_2_-coated NaY powders obtained earlier were compacted under a uniaxial force of 5,000 tons to form a pellet with a diameter of 13 mm and a thickness of ∼1 mm. The pellet was calcined at 600 °C for 2 h. The gold paste was painted on the pellet surface according to the electrode configurations shown in Figure 1(a). These pellets were then heated to 600 °C for 2 h to burn out the temporary binders and increase the adhesion between the gold and the surface of the pellet. Similarly, the electrode structure of the membrane type sensors is illustrated in Figure 1(b). The as-prepared zeolite Y thin films were painted with two gold lines and fired at 600 °C for 2 h to burn the organic binders in the gold paste. Two gold wires served as the leads to the terminals of the impedance spectroscopy apparatus.

The sensing measurement protocol followed the method described in [22]. Briefly, the fabricated sensors were placed in a quartz tube in a programmable high temperature furnace. The gas vapors were introduced by bubbling air through liquid DMMP that was kept at ∼0.5 °C. The gas flow rate was controlled by pre-calibrated digital mass flow controllers (MFC, Sierra) and the concentration was estimated based on the vapor pressure at 0.5 °C. The partial pressure of DMMP in dry atmosphere is around 10 Pa at 0.5 °C [24].

Impedance spectroscopy (Solartron 1260) was employed to generate the cycling excitation voltage with a magnitude of 300 mV. A frequency range from 1 Hz to 10^6^ Hz was conducted during each scan. The impedance analyzer is controlled and the data was analyzed by commercial softwares Zplot and Zview (Scribner Inc., Southern Pines, NC, USA).

## 3. Results and Discussion

### 3.1. Surface Modification of NaY by CeO_2_

**Figure 2.**
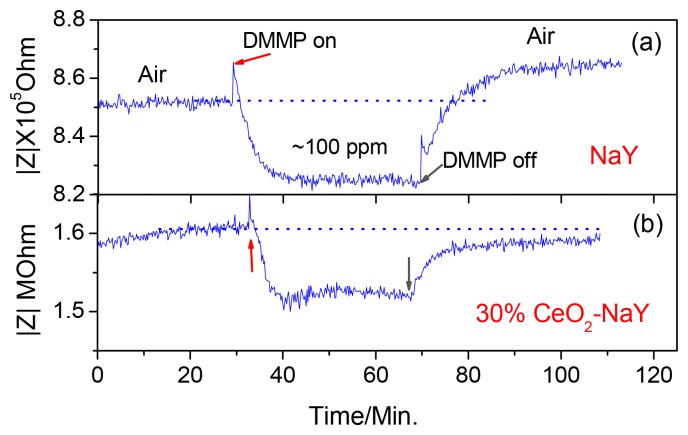
The first-time response of (**a**) NaY and (**b**) 30% CeO_2_ coated NaY to ∼100 ppm DMMP at 320 °C at a fixed frequency of 3,000 Hz.

Figure 2 shows the response curve of a pressed pellet of NaY powder to ∼100 ppm DMMP at 320 °C with a fixed frequency of 3,000 Hz. It takes about 10 min for response/recovery to occur. More importantly, after the sensor is exposed to the DMMP, the impedance of the sensor does not recover back to the starting value and is observed for all samples. An increase of ∼1.3% relative to the initial impedance is calculated for the data in Figure 2. The sensing mechanism involves the transport of sodium cations within the zeolite. The “jumping” rate of the cations is facilitated by the reorientation of the DMMP molecule, leading to the decreased impedance when the sensor is exposed to DMMP. However, it is known that there are residual acidic protons at the surface of the zeolite particles that can bring about decomposition of DMMP. Our hypothesis is that the acid-group induced DMMP decomposition products, such as phosphates can bind the cations and increase the impedance [25]. Based upon this hypothesis, we attempted to neutralize the active proton sites using CeO_2_. Ceria has been employed to modify the surface of the H^+^-mordenite to improve the transformation selectivity of the nephene to 2,6-diisopropynaphthalene [26,27].

**Figure 3.**
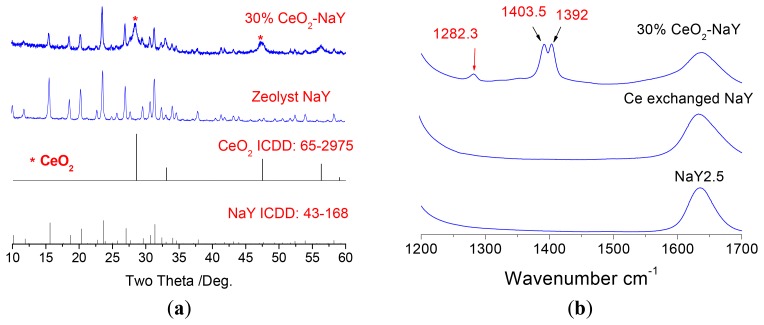

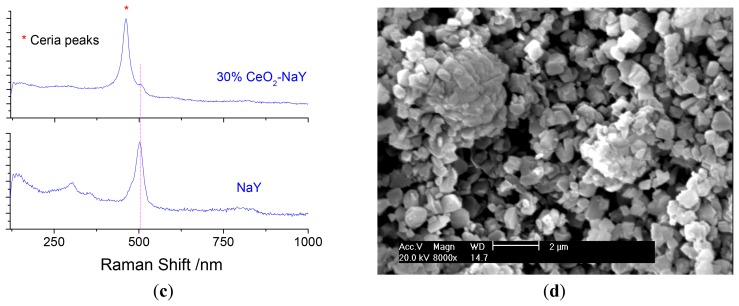
(**a**) XRD patterns; (**b**) Diffuse reflectance infrared spectra; (**c**) Raman spectra and of the various CeO_2_-coated NaY and NaY samples, and (**d**) SEM image of the top surface of the 30% NaY-CeO_2_ pellet after firing at 600 °C for 2 h.

Ceria deposits on the surface of NaY was done using ethanolic cerium nitrate solution followed by calcination. The use of the ethanolic solution precludes ion-exchange into the zeolite. Three loading levels of CeO_2_ were chosen 10, 30 and 50% by weight. Optimal sensing data was obtained with 30% CeO_2_/NaY and forms the focus of discussion. Figure 3a shows the powder X-ray diffraction patterns of the NaY and the 30% NaY-CeO_2_ composite after firing at 600 °C for 2 h, and indicates that with the CeO_2_ coating, the NaY structure was not destroyed. This was true at all loading levels of CeO_2_. Figure 3(b) shows the vibrational spectra of the NaY and CeO_2_ coated NaY samples. As observed from the IR spectra shown in Figure 3(b), carbonates (IR bands at 1,282, 1,403, and 1,392 cm^−1^) do not appear in NaY and Ce ion-exchanged NaY samples, but are present on the coated sample. The formation of cerium carbonate is consistent with the basic character of ceria. Similarly, the CeO_2_ peaks (band at 460 cm^−1^) in the Raman Spectrum Figure 3(c) are also observed for CeO_2_-coated NaY. Figure 3(d) shows SEM image of the top surface of the pressed 30% NaY-CeO_2_ pellet after firing at 600 °C. The pellet (*i.e.*, the sensing body) indicated a porous microstructure between the zeolite particles, a desirable result for gas sensors. These structural studies show that the CeO_2_ was successfully coated on the surface of the zeolite particles, is consistent with the literature [26,27].

### 3.2. Impedance Changes with Ceria-Coated Zeolite Y Pellet-Type Sensors

**Figure 4.**
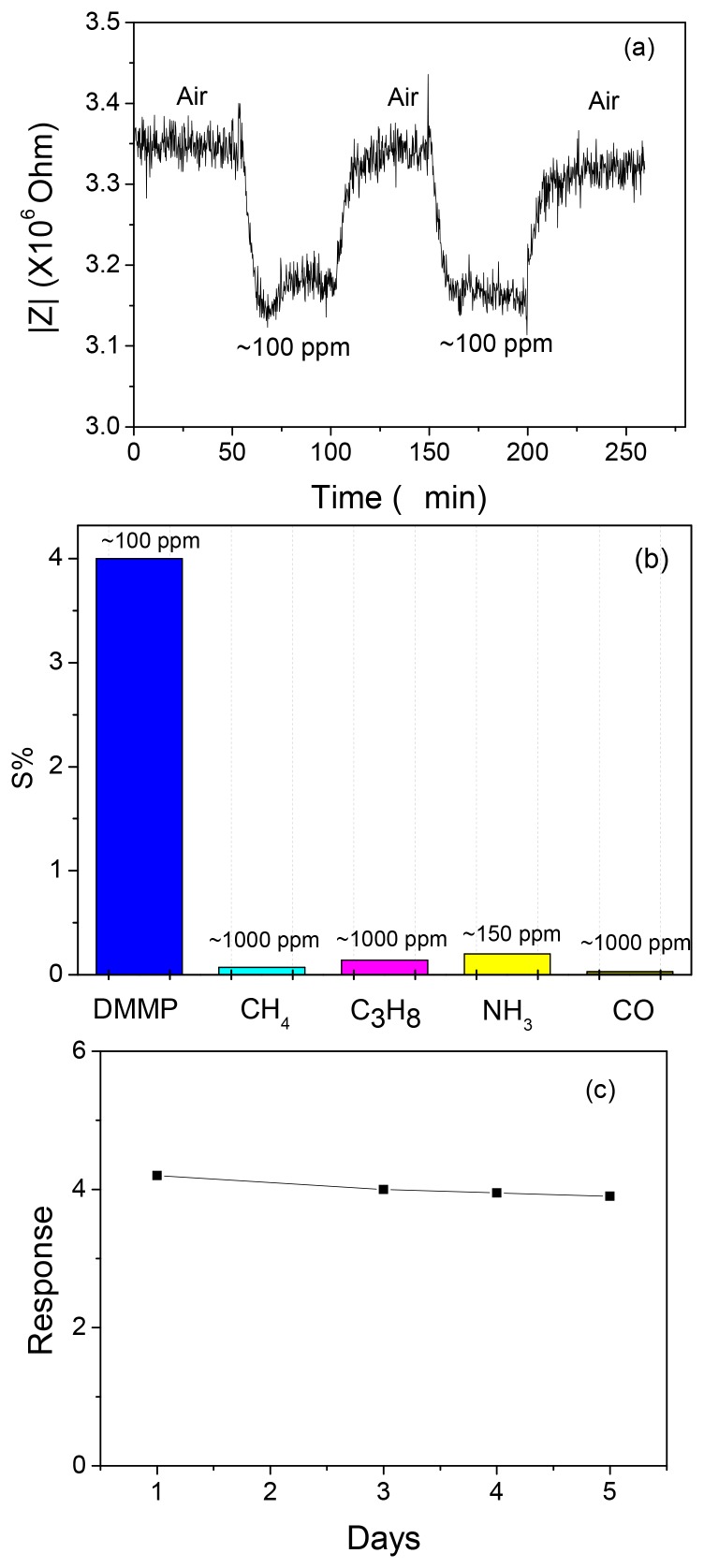
(**a**) Signal reproducibility; (**b**) selectivity and (**c**) long-term signal stability of the 30% CeO_2_-coated NaY based sensors to ∼100 ppm DMMP at 320 °C with a fixed frequency of 3,000 Hz.

With pellets prepared from the zeolite powders, the sensing properties to DMMP at an elevated temperature were investigated. Figure 2(b) shows the first-time response curves of the NaY sample coated with CeO_2_ to ∼100 ppm DMMP at 320 °C with a fixed frequency of 3,000 Hz. Similar results were obtained with the 10% CeO_2_ coating, whereas the 50% CeO_2_ coated NaY sensor indicated an increase and impedance was unchanged even after the removal of DMMP from the gas stream (data not shown). The change in baseline (before and after exposure to DMMP) was optimal with the 30% CeO_2_-coated NaY sample (about 0.2%), and it also showed a faster response time (∼5 min) compared with the NaY pellet. It should be noted that the Ce ion exchanged zeolite Y showed almost no response to DMMP (data not shown). This indicates that for the 30% NaY-CeO_2_ composite, the improvement in the response features such as shorter response times and full recovery is attributed to the CeO_2_ coating instead of the Ce ions, even if any, inside the “supercages”. The reproducibility, selectivity and the five-day stability of the response of the 30% CeO_2_-coated NaY based sensor to DMMP is shown in Figure 4(a–c), respectively. Good signal reproducibility and a reasonably stable response after five days of operation was observed. The sensor also showed excellent selectivity to possible intereferents such as hydrocarbons, CO and ammonia, which is similar to that of the pure NaY reported previously [22]. It appears that ceria coating can successfully improve the recovery feature of the sensors, leading to desorption of DMMP and full recovery of baseline after first-time exposure to DMMP. It has been pointed out by Kim *et al.* [26,27] that rare-earth oxides have amphiphilic properties, *i.e.*, they have both weak Lewis acidity and basicity. It is the basicity of ceria that is being exploited here to reduce the Bronsted acid activity at the external surface of zeolite. When the ceria percentage increased to 50%, it is suspected that too much ceria is blocking the analyte transport, and DMMP is not penetrating into the zeolite.

### 3.3. Zeolite Y Membranes as Impedance-Based Sensors

**Figure 5.**
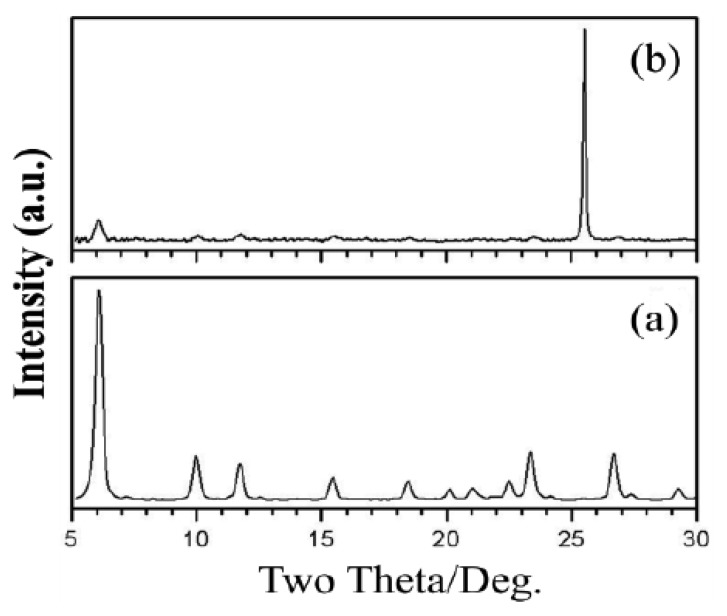
X-ray diffraction patterns of (**a**) zeolite Y standard and (**b**) zeolite membranes.

**Figure 6.**
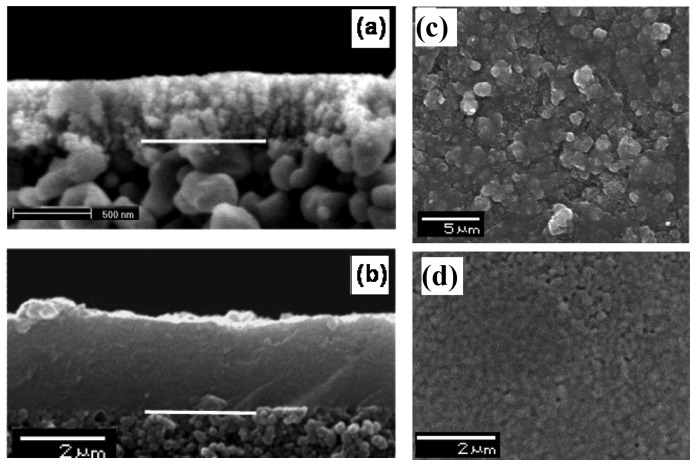
SEM images of cross-sections of (**a**) the seeded thin film and (**b**) after the zeolite film growth, and of the top surfaces of (**c**) the seeded thin film and (**d**) after the zeolite film growth (white bar shows demarcation between zeolite membrane and support).

The second system that we investigated was zeolite Y membranes. Our hypothesis was that the increased length scale of the zeolite in the membrane as compared to the zeolite powder will increase the sensitivity. A seed layer of nanozeolite Y was deposited on an alumina support and zeolite membranes grown from the seeded layer. Figure 5 shows the powder diffraction of the zeolite membrane and indicates the formation of zeolite Y. Figure 6 shows the SEM of the seeded layer and the zeolite membrane grown from the seeded layer, with a thickness of 3 μm.

**Figure 7.**
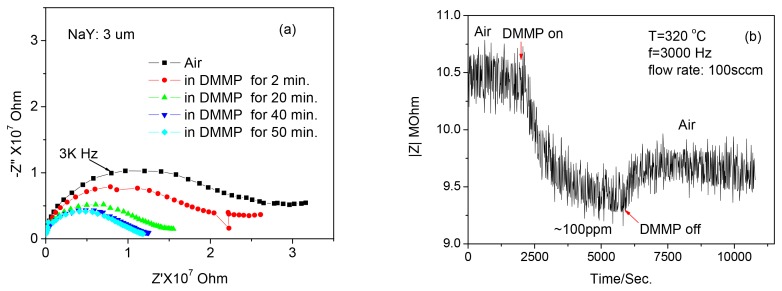
(**a**) Change of the impedance spectra and (**b**) the response curve of the 3 μm thick zeolite film type sensors at a fixed frequency of 3 kHz as a function of time to ∼100 ppm DMMP at 320 °C.

The impedance spectra (1–10^6^ Hz) of the membrane zeolite Y to 100 ppm DMMP at 320 °C as a function of time are shown in Figure 7(a) and the response/recovery of the sensor at a fixed frequency of 3,000 Hz is shown in Figure 7(b). It takes around 40 min for the sensor response to become saturated.

**Figure 8.**
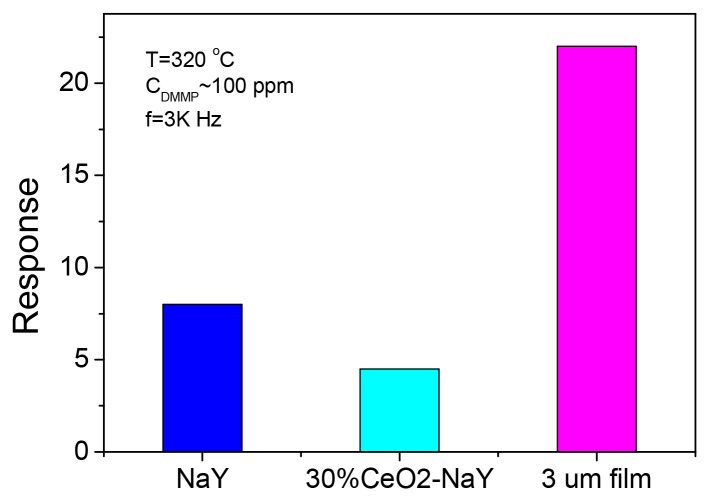
Comparison of the response of the three sensors including NaY, 30% CeO_2_-coated NaY and 3 μm thick NaY film to ∼100 ppm DMMP at a fixed frequency of 3,000 Hz at 320 °C.

A comparison on the response of the three sensors including pure NaY based, 30% CeO_2_ coated NaY and 3 μm film to ∼100 ppm DMMP at 320 °C with a fixed frequency of 3 kHz is illustrated in Figure 8. The 30% CeO_2_-coated NaY sample shows a lower response to DMMP although it had improved the response features and suppressed the incomplete recovery relative to NaY based sensor. The membrane type sensor displayed the largest response among these three samples. The increased response by a factor of 3 of the zeolite membrane as compared to the pressed pellet of zeolite Y is quite remarkable. One possibility is that the motions of the extra framework cations closer to the surface of the pressed pellet samples are relevant to the sensing and DMMP-cation interaction facilitates this motion. With the membrane the intragrain cation motion is relevant, and possibly with a higher activation energy. Thus, for the membrane, the DMMP-cation interaction within the grain brings about a larger decrease in impedance because of the higher activation energy to cation motion [28]. The poor recovery time of the zeolite film as compared to pressed pellets is also consistent with the near-surface mechanism in pellets and penetration of DMMP deeper into the grain in the membrane. Near-surface adsorption leads to better response/recovery times, whereas penetration into the grain slows both the recovery and response. Nevertheless, these observations encourage us to conduct further systematic work to investigate the sensing properties of the membrane type zeolite sensors.

## 4. Conclusions

The sensors using pressed pellets of ceria surface (30% wt) modified zeolite Y showed shorter response/recovery times as well as full recovery of the baseline upon exposure to DMMP. We attribute this effect to the ceria layer on the surface of the zeolite as effectively removing the active protons on the surface of the zeolite and at the same time, does not ‘block’ the windows of the “supercages”. Zeolite membrane based sensors show higher response relative to the pressed pellets of NaY and CeO_2_-coated NaY. However, the membrane-type sensors exhibit longer response/recovery times, possibly related to diffusion of DMMP within the microstructure of zeolite membranes. In the case of pressed pellet type sensors, we propose that the near-surface regions of the zeolites are responsible for the signals.
